# Supplementary material for: Identification and Characterization of Three Heat Shock Protein 90 (Hsp90) Homologs in the Brown Planthopper
Source: Genes (Basel). 2020 Sep 12;11(9):1074. doi: 10.3390/genes11091074 (PMC7563703; doi:10.3390/genes11091074)
Supplement: Supplementary file 1 [file genes-11-01074-s001.pdf]

Table S1. The main primers used in this study.

Primer name	Sequence (5'-3')*	Length (bp)	Purpose
NlHsp90-RNAi-F	T7-TCCGCTGGAGGTTCGTTTA	477 bp	*NlHsp90* dsRNA synthesis
NlHsp90-RNAi-R	T7-CTCCCAGTCGTTGGTGAGC		
NlGRP94-RNAi-F	T7-CCAGTTCGGTGTTGGATT	327bp	*NlGRP94* dsRNA synthesis
NlGRP94-RNAi-R	T7-TCTTCGGATTTGGCTTCT		
NlTRAP1-RNAi-F	T7-CCGAGACCAAGCCAGACAT	441bp	*NlTRAP1* dsRNA synthesis
NlTRAP1-RNAi-R	T7-AGTAGGGCGAACTCAAAGC		
QNlHsp90-F	CACAACGACGATGAGCAAT	192bp	qRT-PCR for *NlHsp90*
QNlHsp90-R	GGGGTAGCCGATGAACTG		
QNlGRP94-F	GGAACTATCGCCAAATCG	193bp	qRT-PCR for *NlGRP94*
QNlGRP94-R	CAGCGTCAGACTCCCAGA		
QNlTRAP1-F	CTCCAACCTGGGCACAAT	106bp	qRT-PCR for *NlTRAP1*
QNlTRAP1-R	GAAGCCGACTCCGAACTG		
QNl18S-F	CGCTACTACCGATTGAA	132bp	qRT-PCR for *Nl18s*
QNl18S-R	GGAAACCTTGTTACGACTT		

* T7, 5′-TAATACGACTCACTATAGGGAGA-3′.

Table S2. The 34 Hsp90 homologs derived from 18 species.

	Species	GenBank accession number
cytosolic Hsp90(Hsp90, Hsp83)	*Nilaparvata lugens*	MT221445
	Laodelphax striatellus	AHB63833.1
	Sogatella furcifera	AFK64820.1
	Bemisia tabaci	ACH85198.1
	Halyomorpha halys	XP_014284945.1
	Locusta migratoria	AAS45246.2
	Zootermopsis nevadensis	XP_021934936.1
	Apis mellifera	XP_006571335.2<br>NP_001153536.1
	Tribolium castaneum	ABR32189.1
	Manduca sexta	XP_030032643.1
	Bombyx mori	NP_001036876.1
	Chilo suppressalis	BAE44307.1
	Papilio xuthus	KPJ05576.1
	Drosophila melanogaster	NP_001261362.1
	Anopheles gambiae	sp|Q7PT10.3|

ER-based	Hsp90(endoplasmin, Gp93)	*Nilaparvata lugens*	MT221444
		Bemisia tabaci	XP_018898642.1
		Halyomorpha halys	XP_014277591.1
		Locusta migratoria	ACS75351.1
		Zootermopsis nevadensis	XP_021916906.1
		Apis mellifera	XP_395614.3
		Tribolium castaneum	XP_971540.1
		Manduca sexta	XP_030029527.1
		Bombyx mori	NP_001266403.1
		Papilio xuthus	KPJ01679.1
		Drosophila melanogaster	NP_651601.1
		Anopheles gambiae	XP_321706.5
mitochondrial TRAP1		*Nilaparvata lugens*	MT221446
		Drosophila melanogaster	NP_477439.2
		Bombyx mori	AFG30049.1
HtpG		*Escherichia coli*	VWQ01303.1
		Pseudomonas aeruginosa	AUA78187.1
		Beauveria bassiana	KGQ13800.1
